# Implementing demand side targeting mechanisms for maternal and child health-experiences from national health insurance fund program in Rungwe District, Tanzania

**DOI:** 10.1186/s12992-016-0180-x

**Published:** 2016-08-02

**Authors:** August Kuwawenaruwa, Gemini Mtei, Jitihada Baraka, Kassimu Tani

**Affiliations:** Ifakara Health Institute, Plot 463, Kiko Avenue Mikocheni, P.O. Box 78 373, Dar es Salaam, Tanzania

**Keywords:** Insurance, Demand-side-financing, Equity, Individual and geographical targeting mechanism, Tanzania

## Abstract

**Background:**

Low and middle income countries have adopted targeting mechanisms as a means of increasing program efficiency in reaching marginalized people in the community given the available resources. Design of targeting mechanisms has been changing over time and it is important to understand implementers’ experience with such targeting mechanisms since such mechanisms impact equity in access and use of maternal health care services.

**Methods:**

The case study approach was considered as appropriate method for exploring implementers’ and decision-makers’ experiences with the two targeting mechanisms. In-depth interviews in order to explore implementer experience with the two targeting mechanisms. A total of 10 in-depth interviews (IDI) and 4 group discussions (GDs) were conducted with implementers at national level, regional, district and health care facility level. A thematic analysis approach was adopted during data analysis.

**Results:**

The whole process of screening and identifying poor pregnant women resulted in delay in implementation of the intervention. Individual targeting was perceived to have some form of stigmatization; hence beneficiaries did not like to be termed as poor. Geographical targeting had a few cons as health care providers experienced an increase in workload while staff remained the same and poor quality of information in the claim forms. However geographical targeting increase in the number of women going to higher level of care (district/regional referral hospital), increase in facility revenue and insurance coverage.

**Conclusion:**

Interventions which are using targeting mechanisms to reach poor people are useful in increasing access and use of health care services for marginalized communities so long as they are well designed and beneficiaries as well as all implementers and decision makers are involved from the very beginning. Implementation of demand side financing strategies using targeting mechanisms should go together with supply side interventions in order to achieve project objectives.

## Background

Low and middle income countries have adopted targeting mechanisms as a means of increasing program efficiency in reaching marginalized people in the community given the available resources [1–3]. A targeting mechanism is a policy option of concentrating the benefits of an intervention on a pre-identified specific group [4]. This has been widely applied in health care interventions in order to extend health care services to the vulnerable groups in the community. These targeting mechanisms include individual [5, 6], geographical [7] and self selection [2, 3]. Implementation of such mechanisms require actors who will be responsible for the implementation of the targeting method and the subsequent implementation of the intervention [1]. The actors/implementers could either be from the national level, regional, district, facility or community level based on the program design and overall purpose.

**Fig. 1:**
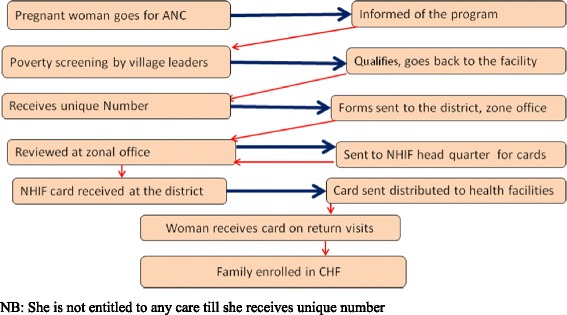
Individual Targeting. The figure shows complexity involved in obtained the MCH insurance card during individual targeting in the study area

**Fig. 2:**
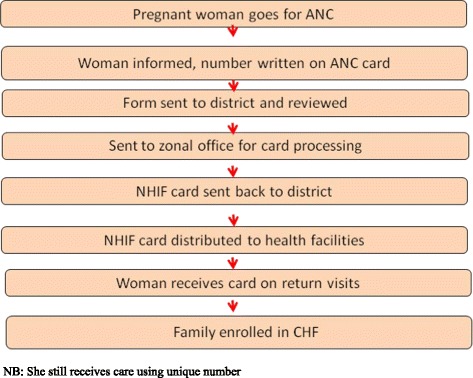
Geographical Targeting. The figure shows how women obtained the MCH insurance card during geographical targeting in the study area. The process was less complex compared with individual targeting

In 2010, Tanzania’s National Health Insurance Fund (NHIF) and German Development Bank (KfW) used geographical and individual targeting mechanisms in Rungwe district to pilot a social health insurance program (MCH insurance card) for poor pregnant women. Women were identified using a score card which had eight components that related to housing characteristics, remoteness from health providers, water source, fuel for cooking, toilet facility, income, food security, and number of dependents [8]. During antenatal care (ANC) visits women were informed of the program by the provider; thereafter they were given registration form and the score card. They go back to the village and presented the card to the village leaders who assessed them based on the screening criteria, then the woman returned the score card to provider. Scores for each criteria ranged from 1 to 3, addition of the score resulted into a minimum of 8 score and a maximum of 24 scores. Women scoring 8 – 18 qualified for the program (termed as “poor”) and were given the MCH insurance card and used it to access care from accredited health care facilities [8]. Under geographic targeting all pregnant women in the geographic areas of the project qualified for the card. Health care providers enrolled pregnant women at the facility after informing them about the program. A unique number was written on top of the ANC card. Registration forms were then taken to the district level and later on submitted to the regional office for entry into a computer system. Cards were processed at the regional level and sent back to the district where they are distributed to the facilities. Women received the cards as they came for subsequent ANC visits to the facility. Figures 1 and 2 show steps which were required to obtain a card under the individual and geographical targeting respectively.

Evidence shows that many countries have been using such targeting mechanisms to accelerate access and use of maternal and child health care [9, 10]. In some countries, interventions which were implemented using targeting mechanisms had a positive impact [11, 12], while others had negative or no impact [13]. For example a study conducted in Uganda showed that the program resulted into increase in number of safe deliveries in the intervention area immediately jumping from <200 deliveries/month to over 500 deliveries/month [12] and revenues had been used to obtain needed supplies to improve quality and to pay health workers, ensuring their availability at a time when workloads are increasing [12, 14, 15]. A recent study conducted by Ifakara Health Institute on a similar project showed that the program impacted few outcomes (mainly out of pocket payment), and no effect was observed on the quality of care and equity [16]. Proper implementation of such targeting mechanisms has great potential for achieving the policy objectives and enhancing program efficiency [4, 11, 17].

Design of targeting mechanisms in the study region has been changing over time and it is important to understand implementers’ experience with such targeting mechanisms. At this point in time, however, in Tanzania there is limited evidence on implementers’ and decision-makers’ experience with targeting mechanisms, the implementation process, and results of switching between the two mechanisms. The aim of this study was to examine the implementer’s experience in the process of implementing targeting mechanisms since such mechanisms impact equity in access and use of maternal health care services.

## Methods

### The study setting

The study was conducted in Rungwe district in Mbeya Region. According to a 2012 census the district has a population of about 339,157 (male 161,249 and 177,908 female) [18]. The population density is high in Rungwe district (153 compared to the national average 51 people per square kilometre) and the main economic activity is agriculture [18]. Out of 10 district councils in Mbeya, Rungwe was the only district that had implemented the intervention using the two targeting mechanisms (individual and geographical targeting), while the other districts had adopted a geographical targeting mechanism. It thus offered not only the opportunity to describe the implementer’s experiences with the current geographic targeting strategy, but also to explore the implementer’s attitudes toward both strategies and the reasons for abandoning individual targeting.

### The study design

The study adopted case study methodology, an empirical inquiry that investigates a phenomenon within its real life context [19]. Implementation of maternal and child health care using targeting mechanisms is a complex, context dependent process. The case study approach was thus considered the appropriate method for exploring implementers’ and decision-makers’ experiences with the two targeting mechanisms.

### Data collection techniques

The study used in-depth interviews in order to explore implementer experience with the two targeting mechanisms. Because the implementation of the MCH insurance card program involved stakeholders at the national level (NHIF and GFA consultant), regional level (NHIF zone office, Regional medical Office), and district level (NHIF/CHF coordinators, and Council Health Management team), as well as health care providers, it was necessary to collect data at all these levels. Study participants were purposively selected from public health care facilities, district and regional level health authorities, and NHIF headquarters (Table 1). The health system in Tanzania assumes a pyramidal pattern of a referral system starting from village health service which is the lowest level of health care delivery in the country followed by dispensary services which cater for between 6000 and 10,000 people and supervise all the village health posts in its ward. Next level of care is the health centre which is expected to cater for 50,000 people, followed by district hospitals, regional hospitals and lastly referral/consultant hospitals. Ongoing health care financing reforms intends to increase access and use of health care services to the people by building a dispensary in every village and a health centre in every ward [20]. Rungwe district had only one district hospital and two public health centres. All of them were included in the study and four dispensaries were selected based on physical accessibility, experience with the targeting mechanisms, and being served by a health centre. Thus, a total of 7 health care facilities were included in the study. In total, 10 in-depth interviews (IDI) and 4 group discussions (GDs) were carried out by 2 research scientists and 2 field assistants in September 2014 (Table 1).

**Table 1:** Qualitative interview sampling for data collection

Location	People to be interviewed
Mbeya Regional Hospital	□ In-charge at RCH department (In-depth Interview)
Mbeya Regional	□ Regional Medical Doctor (In-depth Interview)
District Hospital	□ In-charge at RCH department and assistants ( Group Discussion)
Ikuti Health Centre	□ In-charge at RCH department and assistants (Group Discussion)
Masukulu Health Center	□ In-charge at RCH department and assistants (In-depth Interview)
Kyobo Dispensary	□ In-charge at RCH department and assistants (In-depth Interview)
Kiwira Dispensary	□ In-charge at RCH department and assistants (In-depth Interview)
Bujela Dispensary	□ In-charge at RCH department and assistants (In-depth Interview)
Ilalambwe Dispensary	□ In-charge at RCH department and assistants (In-depth Interview)
Rungwe District	□ District Medical Officer (DMO)/RCH Coordinator/NHIF/CHF Coordinator ( Group Discussion)
Mbeya -Regional/Zone Level	□ NHIF Officer (In-depth Interview)
Mbeya -Regional/Zone Level	□ NHIF Zone Coordinator ( Group Discussion)
National level	□ NHIF Head quarter (In-depth Interview)
National level	□ Meda –Consulting firm representative (In-depth Interview)

Interview guides were developed and contained a range of topics related to the experience with individual targeting, decision to change, experience with geographical targeting, and recommendations about targeting mechanisms. Interview guides were prepared in English and subsequently translated into Kiswahili by the bi-lingual research scientists and research assistants, who also conducted the interviews. Interviews were conducted in pairs: one research scientist facilitated the interview while a field assistant was taking notes. All interviews were also digitally recorded, and the audio files were transcribed and translated by a research assistant. Subsequently, the researchers cross-checked the audio files and transcripts for data quality assurance.

### Data analysis

A thematic analysis approach was adopted. Two research scientists read each transcript independently and developed a final code book. A brief discussion was held by the researchers and to determine the final themes. The team worked together and coded a few transcripts together. The remainder were coded independently by each of the research scientists. At the end the team reconvened and discussed the coded scripts. Data were analyzed using Nvivo 10 software.

## Results

Results have been organized into three major themes: individual targeting, experience with switching targeting mechanism and geographical targeting. Under individual targeting we have four main subthemes these includes experience with program management, experience with program engagement, experience with the screening process and bureaucracy in accessing the card. Experience with switching targeting mechanism has been categorized into three subthemes these includes engagement in decision making, lessons from other districts and experience with the communication process. Results on geographical targeting have been categorized into three subthemes which include increase in facility resources, insurance coverage and health service provision.

### Individual targeting

#### Experience with program management

According to those interviewed, MCH insurance card program activities were executed using the existing government structures. No new staff was employed to handle the activities and no additional payments were made to the existing staff. For example at the district level, district officials were expected to conduct sensitization about the MCH insurance card, conduct job training for the health care providers, handle client complaints, supervise the health workers at facilities, ensure that screening forms were available and were submitted to the respective levels as required, prepare and distribute cards, and act as a link between the zone, district office, and health facilities. At the village level, the village leaders were supposed to undertake screening of potentially eligible women using the eight criteria’s in the score card.

*“……as a focal person at the district level I was supposed to handle client’s complains, conducting community sensitization, conducting supportive supervision in the facility to oversee overall implementation of the program, taking the screening/registration forms from village executives and healthcare providers to the district, also submitting the forms to the regional level*…” (GD, with district level implementer)

“….*because it’s the same person fulfilling same tasks……….for example I was supposed to continue with my normal duties as a …..…….while at the same time fulfilling MCH insurance card tasks which were not within my normal routine, so the time which I was supposed to keep records, review inventories, requisition of supplies and supervision of medical supplies, part of the time was being used on* MCH insurance card *project in one way or another I can say fifty percent of my tasks were affected*….” (GD, with district level implementer)

#### Experience with program engagement

Most of the respondents from the zone and district level implementers had a positive perception of the program and perceived that it was initiated to support women and improve health care services in the region; however, many challenges in the process of implementation were reported. For example, it was reported that in some cases, the individual targeting approach had created a sense of stigmatization. Village executives were supposed to justify that beneficiaries were poor based on the screening criteria, but the community perceived that the need to obtain such justification created stigmatization in the community, causing women to not enrol in the program. The quote below testifies that.

*“..with individual targeting it was viewed that providers were discriminating people and were not doing fairness to some women, those with insurance were being treated as those insured by the National Health Insurance Fund, but the one who had no MCH insurance card was supposed to pay from the pocket..”* (IDI with health care provider)

Furthermore, most of the respondents interviewed from the district level supported the program; however there was a lot of paper work, bureaucracy, and some of the women were not registered because of the whole process in which one was supposed to be confirmed to be poor by village executives,

*“…….It is a good program but has a long process for a woman to get a health insurance card and this lead to some of the women to not be registered,because of missing photographs and sometimes termed to be not qualified as poor…..*”(GD, with district level implementer)

#### Experience with the screening process

According to zone and district level implementers, the process resulted in falsification of the real poverty condition of women, leading to the enrolment of non-poor women. Village executives used the opportunity of screening to enrol their relatives and friends, which also resulted in complaints. Targeted women were not enrolled because of favouritism of some of the village leaders who were given mandate/autonomy to conduct screening.

*“Women were complaining that some of the village executives were recruiting even those who have good houses, those who have money to earn a living, their relatives and leave out those who were real in need of the free health services”*(GD with Health care provider)

Centrally the regional implementers, reported that on the administration issues, there were challenges with the screening forms, as they were filled incorrectly, as stated in the quote below.

*“….one of the challenge was related to the screening process as most of the forms were not returned back from village executives, in case she had a lot of other tasks to handle, the forms were not returned on time and could not be sent to the regional level and headquarter, at times they were sent but too late so some of the services which were intended for the women before delivery were not rendered…”* (GD, district level implementer)

A related challenge that implementers faced during individual targeting was informal payments. During the screening process, some village leaders requested money from the women before signing the forms.

“…..*one of the challenges with individual targeting was on the role of village executives, as some of them were using the screening forms as business, a woman was supposed to pay some money to get the signature; in addition to that the forms did not portray the reality of someone’s economic status*….” (GD, with zone level implementer)

### Bureaucracy in accessing the card

Difficulties were also encountered in obtaining the photo necessary for the identity card under the individual targeting scheme. A photographer in the village was contracted to take photographs; however, this activity created challenges for both the photographer and the potential beneficiary. It was difficult to arrange to have multiple women photographed at a single site and time, and there were also problems with the photographer not being available as scheduled and with payment arrangements/mechanisms. Payments for the photographer were through cheques and were delayed for a number of months. Most of the respondent highlighted that:

*“……it had a long process as sometimes women were organized but the photographer was not available, sometimes the photographer was around but women were not present……..*.” (GD with district level implementer)

Interviews also revealed that during individual targeting, women could not access health care services until they received the insurance card. At the same time, card processing took a long time, and women sometimes delivered before receiving their cards. This was perceived as wastage of the resources because of costs incurred in the whole process of identifying eligible women.

*“……in case there have been delays of the identity card while delivery date is due, the card become useless while costs have been incurred in screening, photographing and preparing the cards….”* (GD with district level implementer)

### Experience with switching targeting mechanism

#### Engagement in decision making

According to regional implementers, the decision to change the program was based on complaints from women within the catchment population as they came to seek health care services. They complained to health providers that they had not been enrolled even though they were eligible:

“….*as I said before some of women were being charged by the village executives and sometimes executives were not in the offices as a result forms were not signed on time*…..” (GD, regional level implementer)

In addition to the complaints, national level and regional managers visited the districts and discussed the progress of the project with council health management teams. They collected some suggestions which led to the shift from individual to geographical targeting.

“…..*I remember as they came to give us information they inquired about the challenges which we had been facing, they questioned us, as we explained the challenges they asked some questions, and we explained the challenges later on they decided to make it geographical*…” (GD, with district level implementer)

### Lessons from other districts

According to the district level implementers, the successes observed in areas which had implemented geographical targeting influenced the decision to switch from individual to geographical targeting. That is, more women in areas with geographical targeting had been enrolled in the program than women in areas with individual targeting. Geographical targeting had fewer complexities, such as screening and photographing, than individual targeting, which had cost and time implications.

*“….it was perceived that program had success in the districts which had implemented geographical targeting as more women were enrolled…..for example they were saying a facility which was in a certain village, women around the facility catchment area had same economic status so any women who was pregnant has to be given a form……”* (GD, with district level implementer)

Furthermore, unspent funds influenced the decision to adopt geographical targeting and grant a one year extension of the program up to December 2015 as resources were still available as the program neared its planned end date.

“…*we were thinking of the costs involved when switching from individual to geographical…but we were told by national level managers that the amount of funds which had been used was small compared with the amount which was budgeted*…..”(GD, with zone level implementer)

### Experience with the communication process

The majority of the implementers and health care providers reported that they received verbal notification from their superiors that they should no longer enrol women on an individual basis using the poverty screening. The decision-making appeared to have been done centrally. Information was sent from the NHIF head office to the zone office, and managers at the zone office conveyed information to the district medical officers at the respective districts in the region. District officials were then responsible to send information about the program to the health facility in-charges.

“….*health care providers were informed about the changes via district medical officers; we communicated with district managers for the districts which were not implementing geographical targeting*…” (GD, with regional implementers)

At the facility level, staffs were responsible for sharing information with the health facility governing committee members and village leaders. Transfer of information from the district to the facility and community level was mainly through telephone and via verbal channels during submission of the claim forms to the district by facility representative. In a number of places, information was conveyed during facility visits by the district and regional implementers.

“…*some people came, I cannot remember from where, as they came they told us there is a project in which pregnant women are supposed to be enrolled in the* MCH insurance card, *moreover they told us that donors had given out funds, and we are supposed to register pregnant women, and will be treated free of charge for the whole duration of pregnancy, delivery and three months after delivery*….” (GD, with Health care provider)

“….*what we did is that, we informed community health workers, so that they could assist us to sensitize women to come and register with the* MCH insurance card *and we were giving them screening forms*… ”(GD, with health care provider)

“…*a person from the district came and told us that there are these and these changes…she said that they have perceived that there were some kind of stigmatization to the women so right now we should start registering all women in the* MCH insurance card *program without looking at income/economic status, no more going to the village executives*….” (GD, with health care provider)

### Geographical targeting

Under geographical targeting, the regional level respondents reported that more women were enrolled in the MCH insurance card program and received care.

“….*with geographical targeting a lot of people have been registered also even if you want to make follow up on the progress of the project it becomes easier, as you go to the facility and review the records on the number of pregnant women who have been registered*….” (GD, with district level implementer)

“….*in general geographical targeting has more than 100 % success compared to the individual targeting*…” (GD, with district level implementer)

In terms of the availability of services, beneficiaries were allowed to access health care at any facility within the intervention region.

“……… *because the government cannot provide everything at the health care facilities, with the use of* MCH insurance card *women can access health care services from private providers, this has reduced congestion in some of the government health facility,* MCH insurance card *gives a woman wider choice of the provider, another thing is that a woman can access medication which are very expensive outside the hospital even if it’s a private hospital, there are certain drugs which are sold more than two hundred thousand (TZS 200,000/~US$90.0), with the insurance program a woman can access the drug*…..” (GD, with district level implementer)

Regional level implementers reported that this resulted in an increase in the number of women going to the regional referral hospital. The reason given for this is that at the hospital level all services are available and beneficiaries learned that the services were available at no cost; they only had to pay for the transport cost to the hospital.

*“If you go there at the ward almost 99 % are* MCH insurance *members, many women are coming, wards are crowded and some women had to sleep on the floor)* (GD, with zone level Implementer)

### Increase in facility resources

Another positive impact was noted in terms of increased facility revenue. With the increased enrolment, providers were able to initiate a number of claims for services after registering women, which increased the amount of revenue to the facility and district. Facilities were able to use the revenue to purchase equipment and fund other health facility needs. In the past, most women delivering in facilities had been exempted from paying and the facilities had to absorb these costs. A majority highlighted that:

*“…now we are sure of having enough drugs in the health care facilities because it will cover a large group of those who are exempted, all the women had exemption, whether you had the ability to render services to them or not, you were forced to treat them for free..”* (GD with zone level implementer)

“…*what I have learned with geographical targeting is that more women have enrolled and benefited from the program, facilities have also benefited as a lot of money comes back to the facility, facility financial resource have increased to the extent of being able to purchase missed equipments and medicines*…” (GD, with district level implementer)

### Insurance coverage

The switch to geographic targeting also resulted in an increase in enrolment in the national insurance scheme. Women who receive the MCH card are entitled to a CHF card for one year together with their partner and up to four children. This card can be used to access health care services from public primary-level facilities. With the greater enrolment following the switch to geographic targeting for the MCH card, coverage of CHF in the district has increased overall from about 3 to 11 %.

One challenge that resulted from the switch in targeting was the increase in workload experienced by health care providers. Health care providers are responsible for registration, education, and submission of claim forms. With the increased number of beneficiaries, this resulted in an increased workload at the facility, district, and regional level. At the same time, however, the number of staff remained the same. When asked about the experience with the geographical targeting, a respondent pointed out that:

*“..we have observed that members have increased about four times compared with individual targeting, this was attributed by the increase in enrolment, registration forms and claim forms leading to the increase of workload to the staffs at all levels..*” (GD, with regional level implementer)

### Health service provision

The increase in work load has been mentioned by health providers and NHIF program implementers at the regional level as a factor that affected the quality of information in the claim forms. The providers mentioned that they have to render services to other clients and at the same time register beneficiaries and complete the claim forms. This resulted in a reduction of quality of services provided and poor attention to the details required for completing the patients’ registration form and claims for service reimbursement.

…..*we have a challenge on how the claim forms are being filled some of the forms are poorly filled, this is something new to the staffs and sensitization has to be done…… you may find a staff was supposed to record this way but she does the other way…..*..” (GD, with regional level implementers)

The NHIF implementers mentioned that there were a number of cases where providers recorded only some of the visits that each patient had made, which resulted in challenges when auditing information at the facility level. There were also reported instances of contradictory information about the same patient being recorded. For example one respondent pointed out that:

*“….a woman is reported to have normal delivery but at the same time she is reported to have caesarean section or same woman was reported delivering at a certain facility but has come to deliver here on referral basis…”* (GD with the regional level implementer)

After the implementation of geographical targeting, card processing was no longer done at the headquarters in Dar es Salaam, but instead at the zone office. This has reduced the time for registration and card processing. Beneficiaries were allowed to access health services before receiving their card as long as they had a unique number written on top of the ANC card.

## Discussion

The case study aimed to examine in-depth the experiences of implementers and decision-makers with maternal and child health demand side targeting mechanisms by studying the implementation of the MCH insurance card program in Rungwe District. More specifically, it describes implementers’ experiences with the current geographic targeting strategy, implementers’ attitudes toward both strategies, and the reasons for abandoning individual targeting. The findings show that implementation of each mechanism has some drawbacks.

It is evident from the findings that although individual targeting mechanisms aimed to enhance financial protection to the marginalized households in the community, it proved challenging to implement. The screening process faced a lot of challenges especially at the community and health care facility level, where some of those who received the insurance card were not the real poor targeted by the intervention. Some implementers demanded payments or enrolled their relatives/friends who were not poor. In many cases, administrative discretion has led to exclusion of people from targeted interventions, and targeting mechanisms which vest a lot of power in the hands of bureaucrats who have been subject to the manipulation of enrolment information [21]. Perhaps lack of incentives to the lower level implementers as the program was implemented within the existing platform contributed to this problem. Other studies have highlighted the importance of using community members to help to determine who is actually poor as they know each other’s resources and needs [22, 23]. It is still questionable whether individual targeting managed to reach the marginalized women in the community, particularly those who were in need of care in the remote areas. According to Hanson et al.*,* benefits associated with taking the targeted benefits does create a sense of stigma to the beneficiaries [22]. Hanson et al., highlighted the problem of not revealing required information and misusing information based on the relationship with the targeted people [22]. A recent study undertaken by the World Bank and the World Health Organization (WHO) on the potential use of the community-directed intervention approach to carry out interventions showed that community level implementers expressed a desire for financial incentives; however the lack of financial incentives did not have a significant effect on their willingness to serve [24]. Incentives must be structured in such a way that there will be transparency and local community accountability in order to ensure effective use of limited resources. For example, Cambodia used individual targeting mechanisms to distribute vouchers for safe delivery to poor pregnant women; findings showed that 24.9 % were identified as eligible for vouchers, while 75.1 % of potentially poor pregnant women were excluded from the program [25]. Ridde et al. argues that use of community-based approach in identifying eligible people who should benefit from exemption program is potentially effective in minimizing errors of inclusion and exclusion [26]. International literature shows that transparency, strength of the local administration, awareness of the program, use of inspection mechanisms [27].

Furthermore, existing bureaucracy impeded the implementation of individual targeting, making the whole process slower than expected. The whole process of screening and identifying poor pregnant women resulted in delay in implementation of the intervention. Efficient collective decision-making mechanisms would reduce bureaucracy in the implementation of demand side financing interventions [28], moreover accountability and transparency of the leaders plays a greater role in the reduction of bureaucracy.

Opportunity cost to those involved in the implementation has been documented as one of the drawbacks with individual targeting [22, 29]. People involved in the targeting mechanisms usually do incur some private costs of travelling to and attending meetings in the intervention areas together with the values of time in participating in the intervention activities [27]. In Cambodia individual targeting was found to induce unnecessary cost to identify the non-poor pregnant women and delay the distribution of vouchers [25].

Additionally, individual targeting was perceived to have some form of stigmatization; hence beneficiaries did not like to be termed as poor. Emerging evidence reveals several key programmatic principals for successful stigma-reduction programs [30]. Successful interventions to reduce stigmatization involve a combination of strategies and approaches, engage a broad range of stakeholders, address intersecting stigmas, and are led by or actively engage communities experiencing stigma [30, 31]. According to Nayar et al., stigma reduction interventions into four types, including (a) information-based approaches, (b) skills building, (c) counselling/support, and (d) contact with targeted groups [30, 31]. Use of social marketing which is concerned with the use of ‘marketing tools, concepts and resources to encourage positive behaviour change among targeted population has been used as a means of reducing stigmatization in the community [32]. Raising awareness by educating beneficiaries, leaders at different levels and health care providers will also help to address the problem of stigmatization.

Evidence from the findings shows that inefficiency in the implementation of individual targeting; complaints from women; lesson from other districts implementing geographical targeting; and unspent funds influenced the decision to adopt geographical targeting. Decision in switching between health care interventions depends on issues of social justice, whether to redistribute resources across society and also whether there are efficiency reasons for providing resource re-distributions [33]. Depending on the available resources then decision can be in favour of individual or geographical targeting.

Implementation of geographical targeting faced some pros and cons. Some of the pros included increased enrolment of beneficiaries in the intervention, increase in the number of women going to higher level of care (district/regional referral hospital), increase in facility revenue and increase in insurance coverage [9, 11, 15]. According to Schmidt et al. demand side financing interventions provides substantial additional funding to facilities however it remains complex to administer such revenue to the extent that it requires a parallel administrative mechanism putting additional work burden on the health workers [34].

The increase in insurance coverage at the intervention (from 3 to 11 %) sites has a substantial increase in overall national insurance coverage. Geographical targeting had a few cons as health care providers experienced an increase in workload while staff remained the same and poor quality of information in the claim forms. Geographical targeting has been widely used in health care interventions because it is simple to administer, has no labour disincentive, is unlikely to create stigma effects and is easy to combine with other targeting methods [35].

Geographical targeting mechanisms usually results in more resources to the providers as more clients seek health care services and provider has to reimbursed for service rendered. Similar to the voucher program which was implemented in Kenya the provider facility managers reported a primary benefit of the program to be the reliable source of revenue it provided [36]. Evidence shows that revenues from the voucher program have been used by facility managers to upgrade the facilities, purchase drugs, supplies, equipment’s, provide incentives to clients such as improve hygiene and nutrition during admission, purchase of beds, provide incentives to providers to deliver quality services and compensate long working hours [9, 12, 36].

Geographical targeting approach requires enough resource for its effective implementation as well as sustainability of the program. For example in Afghanistan there is much more diverse geographically, culturally, and socioeconomically. This indicates that a more diverse targeting strategy, including geographic targeting for high-poverty districts where the cost of individual household targeting does not make sense, may be warranted [37]. Evidence from Ghana showed that the cost to the government of exempting one poor individual from premiums was quite high (US$15.87 to US$95.44) and that means testing (MT) of wealth and geographic targeting (GT) were the optimal mechanisms depending on the setting (poverty level and rural/urban characteristics) [38]. In some cases a mix of individual and geographical targeting might work better in reaching the vulnerable population than the use of single mechanism [39].

### Recommendations

The intervention was implemented following a top-down approach where community/providers and district level implementers received information from the top officials. Communication channels should be strengthened to ensure clear flow of information to and from the community. Feedback is important to the implementers as they will be able to know implementation progress. Future interventions should involve all the implementers from the beginning of the intervention. The choice and decision to implement targeting mechanisms should be based on the accessibility, availability of the resources (human labour and financial), correct identification of the targeted population, and sensitization as well as proper communication at all levels. Furthermore, since the implementation of the intervention was done in parallel with the existing policy of waiver/exemption, there is a need to ensure that such interventions are implemented in such a way they do not create misunderstanding in the system, from both the provider and the user perspectives. Providers should be sensitized on the use of program resources in improving service provision in the facilities. Health care providers should be trained on how to fill registration and claim forms as well as other facility records. One of the main factors contributing to the failure of demand side intervention programs for maternal health programmers is the mismatch between the actual needs of the people and the circumstances in which healthcare is provided [40], there is a need of incorporating such interventions into a coherent district planning and implementation process.

### Study limitation

The study suffered a number of limitations. We were not able to sample village leaders who were involved in the process of screening MCH insurance card beneficiaries. Information from such stakeholders would have added value in terms of their time involvement and informal payments which were experienced during individual targeting. Another limitation was in terms of recall bias, as the study was done one year after decision was made in favour of geographical targeting. We could not manage to sample private health care providers who are also rendering services to the program beneficiaries; their information could have added value to the overall experience with the intervention. However, our findings are useful in the sense that we were able to draw some evidence on the experience with the targeting mechanisms.

## Conclusion

Interventions which are using targeting mechanisms to reach poor people are useful in increasing access and use of health care services for marginalized communities so long as they are well designed and beneficiaries as well as all implementers and decision makers are involved from the very beginning. Implementation of demand side financing strategies using targeting mechanisms should be carefully designed in such a way to ensure minimization of errors of exclusion and inclusion. Furthermore resource allocated for such interventions should be used effectively to minimize wastage of funds as well as time which could be allocated for other activities in order to improve access and use of health care service, with the intension of reaching universal health coverage.

## Abbreviations

ANC, antenatal care; CHF, community health fund; GD, group discussion; GT, geographic targeting; IDI, In-depth Interview; IHI, Ifakara Health Institute; IRB, Institutional Review Board; KfW, German Development Bank; MCH, maternal and child health; MT, means testing; NHIF, National health Insurance Fund; TZS, Tanzania Shillings; US$, United States Dollar; WHO, World Health Organisation

## References

[1] Coady D, Grosh M, Hoddinott J (2008). Targeting of transfers in developing countries: review of lessons and experience.

[2] Handa S, Carolyn H, Nicola, Hypherb, Clarissa T, Fabio S, Benjamin D (2012). Targeting effectiveness of social cash transfer programmes in three African countries. J Dev Eff.

[3] Domelen J. Reaching the Poor and Vulnerable: Targeting Strategies for Social Funds and other Community-Driven Programs. Human Development Network - World Bank; 2007

[4] Meessen B, Criel B. Public interventions targeting the poor: an analytical framework. In: Meessen B, Pei X, Criel B, Bloom G (eds). Health and social protection: experiences from Cambodia, China and Lao PDR. Antwerp: ITG Press; 2008. pp. 263–92. Online at: http://www.strengtheninghealthsystems.be/doc/1/ref%201.22%20public%20interventions%20targeting%20the%20poor%202008shso0263.pdf.

[5] Aryeetey G, Jehu-Appiah C, Kotoh AM, Spaan E, Arhinful DK, Baltussen R (2013). Community concepts of poverty: an application to premium exemptions in Ghana's National Health Insurance Scheme. Glob Health Action.

[6] Collins B (2009). An understanding of poverty from those who are poor. SAGE Publications.

[7] Elbersa C, Fujiic T, Lanjouwd P, Özlerd B, Yine W (2007). Poverty alleviation through geographic targeting: How much does disaggregation help?. J Dev Econ.

[8] Kuwawenaruwa A, Baraka J, Ramsey K, Manzi F, Bellows B, Borghi J (2015). Poverty identification for a pro-poor health insurance scheme in Tanzania: reliability and multi-level stakeholder perceptions. Int J Equity Health.

[9] Ahmed S, Khan MM (2011). A maternal health voucher scheme: what have we learned from the demand-side financing scheme in Bangladesh?. Health Policy Plan.

[10] Bhatia M, Gorter A (2007). Improving access to reproductive and child health services in developing countries: are competitive voucher schemes an option?. J Int Dev.

[11] Bellows B (2013). Increase in facility-based deliveries associated with a maternal health voucher programme in informal settlements in Nairobi, Kenya. Health Policy and Planning.

[12] Ekirapa-Kiracho E, Waiswa P, Rahman MH, Makumbi F, Kiwanuka N, Okui O, Rutebemberwa E (2011). Increasing access to institutional deliveries using demand and supply side incentives: early results from a quasi-experimental study. BMC Int Health Hum Rights.

[13] Bellows N, Bellows W, Warren C (2011). Systematic review: the use of vouchers for reproductive health services in developing countries: systematic review. Trop Med Int Health.

[14] Amudhan S (2013). Effectiveness of demand and supply side interventions in promoting institutional deliveries – a quasi-experimental trial from rural north India. Int J Epidemiol.

[15] Ir P (2010). Using targeted vouchers and health equity funds to improve access to skilled birth attendants for poor women: a case study in three rural health districts in Cambodia. BMC Pregnancy Childbirth.

[16] Borghi J, Kuwawenaruwa A, Binyaruka P, Manzi F. Evaluation of the impact of the nhif mch insurance card on improving access to maternal and newborn care services in Tanzania- (BIMAWAZAZI): Results from the Impact Evaluation of the ‘Helping Poor Pregnant Women Access Better Health Care Project’ in Mbeya Region. Ifakara Health Institute; 2015.

[17] Gopalan SS, Das A, Mutasa R (2014). What makes health demand-side financing schemes work in low-and middle-income countries? a realist review. J Public Health Res.

[18] NBS (2013). Population and housing census; population distribution by administrative areas.

[19] Yin R (2003). Case study research: design and methods.

[20] MoHSW (2007). Primary health services development programme- MMAM 2007 – 2017.

[21] Mkandawire T. Targeting and Universalism in Poverty Reduction: Social Policy and Development, in Programme Paper Number 23. United Nations Research Institute for Social Development; 2005

[22] Hanson K, Worrall E, Wiseman V. Targeting services towards the poor: A review of targeting mechanisms and their effectiveness., in Health System Resource Guide. 2006.

[23] Jaspars S, Shoham J (1999). “Targeting the vulnerable: a review of the necessity and feasibility of targeting vulnerable households”. Disasters.

[24] WHO (2010). Community-directed interventions for priority health problems in Africa: results of a multicountry study. Bull World Health Organ.

[25] Ir P, Horemans D, Souk N, Van Damme W. Using targeted vouchers and health equity funds to improve access to skilled birth attendants for poor women: a case study in three rural health districts in Cambodia. BMC Pregnancy Childbirth. 2010;10:1. 10.1186/1471-2393-10-1 PMC2820432 20059767

[26] Ridde V (2010). Low coverage but few inclusion errors in Burkina Faso: a community-based targeting approach to exempt the indigent from user fees. BMC Public Health.

[27] Slater R, Farrington J (2009). Targeting of social transfers: a review for DFID.

[28] Fozzard A (2001). The basic budgeting problem: approaches to resource allocation in the public sector and their implications for pro-poor budgeting.

[29] Conning J, Kevane M (2001). Community based targeting mechanisms for social safety nets. Social Protection Discussion Paper No 0102.

[30] Nayar US (2014). Reducing stigma and discrimination to improve child health and survival in low- and middle-income countries: promising approaches and implications for future research. J Health Commun.

[31] Stangl AL (2013). A systematic review of interventions to reduce HIV-related stigma and discrimination from 2002 to 2013: how far have we come?. J Int AIDS Soc.

[32] Jacobs B (2012). Addressing access barriers to health services: an analytical framework for selecting appropriate interventions in low-income Asian countries. Health Policy Plan.

[33] Ensor T (2003). Consumer-led demand side financing for health and education: an international review.

[34] Schmidt JO (2010). Vouchers as demand side financing instruments for health care: a review of the Bangladesh maternal voucher scheme. Health Policy.

[35] Coady D, Margaret G, John H (2004). The targeting of transfers in developing countries: review of lessons and experiences.

[36] Njuki R (2015). Does a voucher program improve reproductive health service delivery and access in Kenya?. BMC Health Serv Res.

[37] Steinhardt LC, Peters DH (2010). Targeting accuracy and impact of a community-identified waiver card scheme for primary care user fees in Afghanistan. Int J Equity Health.

[38] Odeyemi IAO, Nixon J (2013). Assessing equity in health care through the national health insurance schemes of Nigeria and Ghana: a review-based comparative analysis. Int J Equity Health.

[39] Axelson H (2009). Health financing for the poor produces promising short-term effects on utilization and out-of-pocket expenditure: evidence from Vietnam. Int J Equity Health.

[40] Malick K, Pison G (2010). Maternal mortality in rural Senegal. The experience of the New Ninéfescha Hospital. Population.

